# Deep learning acceleration of multiscale superresolution localization photoacoustic imaging

**DOI:** 10.1038/s41377-022-00820-w

**Published:** 2022-05-12

**Authors:** Jongbeom Kim, Gyuwon Kim, Lei Li, Pengfei Zhang, Jin Young Kim, Yeonggeun Kim, Hyung Ham Kim, Lihong V. Wang, Seungchul Lee, Chulhong Kim

**Affiliations:** 1 Departments of Electrical Engineering, Mechanical Engineering, Convergence IT Engineering, and Interdisciplinary Bioscience and Bioengineering, Graduate School of Artificial Intelligence, Medical Device Innovation Center, Pohang University of Science and Technology (POSTECH), 77 Cheongam-ro, Nam-gu, Pohang, Gyeongbuk 37673 Republic of Korea; 2 Caltech Optical Imaging Laboratory, Andrew and Peggy Cherng Department of Medical Engineering, Department of Electrical Engineering, California Institute of Technology, 1200 E. California Blvd., MC 138-78, Pasadena, CA 91125 USA; 3 School of Precision Instruments and Optoelectronics Engineering, Tianjin University, 92 Weijin Road, Nankai District, Tianjin, 300072 China; 4 Opticho, 532, CHANGeUP GROUND, 87 Cheongam-ro, Nam-gu, Pohang, Gyeongsangbuk 37673 Republic of Korea

**Keywords:** Photoacoustics, Imaging and sensing

## Abstract

A superresolution imaging approach that localizes very small targets, such as red blood cells or droplets of injected photoacoustic dye, has significantly improved spatial resolution in various biological and medical imaging modalities. However, this superior spatial resolution is achieved by sacrificing temporal resolution because many raw image frames, each containing the localization target, must be superimposed to form a sufficiently sampled high-density superresolution image. Here, we demonstrate a computational strategy based on deep neural networks (DNNs) to reconstruct high-density superresolution images from far fewer raw image frames. The localization strategy can be applied for both 3D label-free localization optical-resolution photoacoustic microscopy (OR-PAM) and 2D labeled localization photoacoustic computed tomography (PACT). For the former, the required number of raw volumetric frames is reduced from tens to fewer than ten. For the latter, the required number of raw 2D frames is reduced by 12 fold. Therefore, our proposed method has simultaneously improved temporal (via the DNN) and spatial (via the localization method) resolutions in both label-free microscopy and labeled tomography. Deep-learning powered localization PA imaging can potentially provide a practical tool in preclinical and clinical studies requiring fast temporal and fine spatial resolutions.

## Abstract (web summary)

Computational strategy based on deep neural networks enables to reconstruct high-density superresolution localization photoacoustic images from far fewer raw image frames.

## Graphical abstract

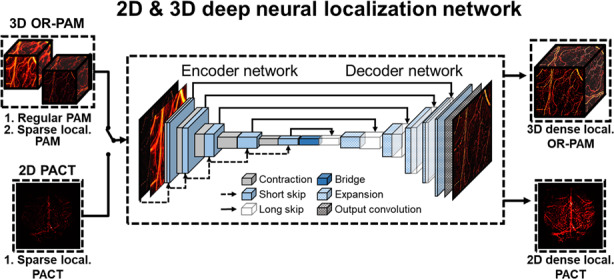

## Introduction

Photoacoustic imaging (PAI), a hybrid imaging technology employing optical excitation and ultrasonic detection, enables multiscale in vivo imaging on scales from organelles to organs^1,2^. PAI generates ultrasonic waves by shining short laser pulses onto biomolecules, which absorb the excitation light pulses, undergo transient thermo-elastic expansion, and transform their energy into ultrasonic waves, called photoacoustic (PA) waves. The induced PA waves are detected by an ultrasound (US) transducer. Depending on the light illumination pattern, the US transducer frequency, and the target imaging depth, the PAI modality is commonly divided into two modes: photoacoustic microscopy (PAM) and photoacoustic computed tomography (PACT). Thus, PAI can provide multiscale and multi-parametric imaging solutions covering resolutions from nano to millimeters at imaging depths from hundreds of micrometers to several centimeters. From single cells to organs in vivo, preclinical PAI systems have been widely used to obtain several types of information: molecular (e.g., biomarkers, contrast agents, and gene expressions), anatomical (e.g., vasculatures, lymphatic networks, and organs), and functional (e.g., oxygen saturation, blood flows, metabolic rates, brain activity, and responses to drug delivery and treatment)^2–14^. PAI has also demonstrated its utility in clinical studies of various cancers, brain diseases, intestinal diseases, and peripheral diseases^15–19^.

Until now, multiscale PAI systems have evolved by improving their spatial and/or temporal resolutions. For example, in optical-resolution PAM (OR-PAM), the temporal resolution has been technically improved by faster scanning and/or laser systems^2^. Theoretically, the lateral spatial resolution is limited by optical diffraction, while the bandwidth of the US transducer determines the axial resolution^20^. Over the last decade, nonlinear PA effects or localization methods, first popularized through single-molecule localization in fluorescence microscopy, such as photoactivated localization microscopy (PALM) and stochastic optical reconstruction microscopy (STORM), have been adapted in OR-PAM to improve its limited spatial resolution^10,21–24^. Notably, a label-free approach to localization OR-PAM using red blood cells (RBCs) has provided superior spatial resolution without any contrast agent^10^. However, obtaining a localized image requires tens of 3D OR-PAM images, which can be infeasible. Inescapably, to significantly improve the spatial resolution, the temporal resolution must be sacrificed. In PACT systems, the temporal resolution is technically restricted by their multi-element US detection and the laser pulse repetition rates, and acoustic diffraction fundamentally limits the spatial resolution^1,19^. Recently, PACT systems using external contrast agents for localization have been actively explored in live animals, in an effort to improve the spatial resolution while maintaining the imaging depth^25–28^. Localizing and superimposing the externally introduced agents in consecutive regular PACT frames enables superresolution imaging beyond the acoustic diffraction limit. However, similar to localization in OR-PAM, localization in PACT requires that hundreds of thousands of images be overlapped, significantly slowing the temporal resolution.

Computational strategies based on a deep neural network (DNN) have proved effective in improving such biomedical imaging modalities as optical microscopy, US imaging, magnetic resonance angiography (MRI), and computed tomography (CT)^29–37^. An especially interesting emerging application minimizes data acquisition times by reconstructing dense data from spatially or temporally undersampled sparse data^30,31^. Here, we introduce DNN-based frameworks to expedite localization-based PAI by reconstructing dense images from sparse information for both 3D label-free localization OR-PAM and 2D labeled localization PACT. Without using any simulated data, we train and validate the DNNs with only in vivo 3D OR-PAM and 2D PACT images. Using only a few frames, our 3D DNN successfully reconstructs 3D dense superresolution OR-PAM images from sparse images, whereas such a dense image generally requires tens of frames to reconstruct. The 2D DNN synthesizes 2D dense superresolution PACT images from sparse images with 12x fewer localized sources than those used for dense images. Our DNN-based localization approach to PAI simultaneously improves the temporal and spatial resolutions, and it could significantly contribute to preclinical and clinical studies requiring fast and fine imaging.

## Results

### Use of a DNN to reconstruct label-free and labeled localization-based superresolution PA images from sparse ones

**Fig. 1:**
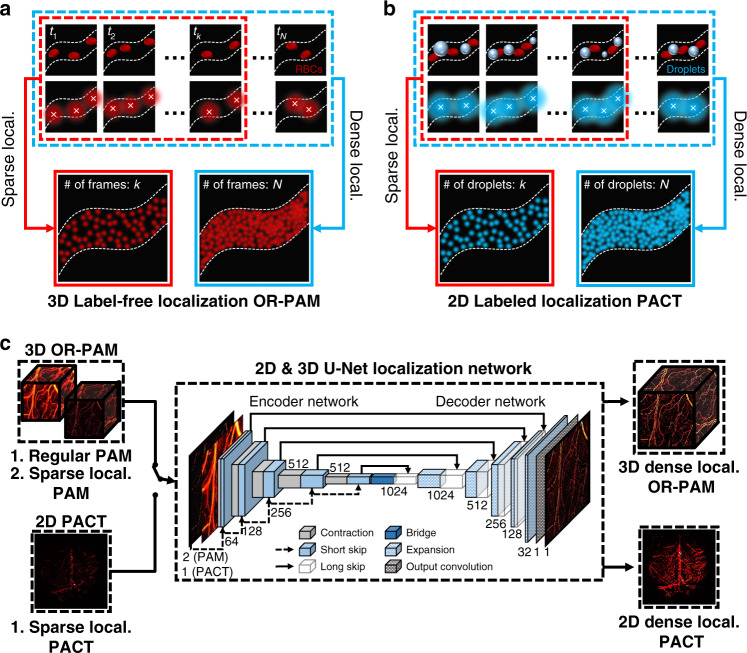
Overview of 3D-2D hybrid deep-learning localization imaging. Acquisition of localization **a** OR-PAM dataset and **b** PACT dataset. Dense localization-based images are generated using N frames in OR-PAM or N dye droplets in PACT. A sparse localization-based image is constructed using k randomly selected images in OR-PAM or k droplets in PACT (k < N). **c** Visual representation of the customized 2D and 3D U-Net generator network architecture. Either 3D sparse localization-based and regular OR-PAM images or a 2D sparse PACT localization image are fed as inputs to the generator. *OR-PAM* optical-resolution photoacoustic microscopy, *PACT* photoacoustic computed tomography, *Sparse local.* sparse localization-based, *Dense local.* dense localization-based

Figure 1 shows an overview of our deep-learning (DL)-based framework that reconstructs a high-density localization-based PA image that includes approximately the same microvascular structural information as a dense localization-based PA image. As ground truth, a high-quality dense localization-based image is created by superimposing *N* frames in OR-PAM or *N* target dye droplet images in PACT. As an input of generators, a poor-quality sparse localization-based image is produced by superimposing *k* (*k*«*N*) frames in OR-PAM or *k* droplets in PACT, which are randomly selected among the *N* frames or *N* droplets (Fig. 1a, b). Due to the difference in the localization processes of label-free OR-PAM and labeled PACT, we reconstructed sparse localization-based images for each case in different ways (Supplementary Text and Figs. S1, S2). For localization OR-PAM, a regular OR-PAM frame was translated into a localization frame (Fig. S1). Then, we randomly selected the translated localization frames to reconstruct sparse localization OR-PAM images. Unlike the OR-PAM localization process, in localization PACT, exogenous absorbers were extracted from regular PACT images. Localized points were then randomly picked to produce a sparse localization PACT image (Fig. S2).

Our framework employs two types of DNNs to cover both label-free localization OR-PAM and labeled localization PACT. Our network for localization OR-PAM contains 3D convolutional layers to maintain the 3D structural information of the volumetric OR-PAM images, and our network for labeled localization PACT has 2D convolutional layers because PACT images are 2D planar images. The DNNs, which are adapted from a pix2pix framework based on a generative adversarial network (GAN) with U-Net^38–40^, learn voxel-to-voxel or pixel-to-pixel transformations from either a sparse localization-based PA image or a dense one. The GAN framework generally consists of a generator network that reconstructs a synthetic image and a discriminator network that outputs the probability that the input image is real or synthetic^39^. Both networks are simultaneously trained by competing against each other, and as training progresses, the distribution of real images is learned to synthesize new images more similar to real ones. In our GANs, generators are designed based on U-net (Fig. 1c), which has recently proven effective for multiscale image learning, especially PA image reconstruction^29,31,38,41^. The generator for 3D OR-PAM images contains 17 3D convolutional layers and roughly 43 million trainable parameters (Table S1). The generator network for 2D PACT images shares the same structure as the 3D network, with 3D operations replaced with 2D operations, and it contains roughly 102 million trainable parameters (Table S1). One structural difference is that we adopted the pixel shuffle operation in the expansion layer for the 2D localization PACT network, because utilizing the transposed convolution operation resulted in unwanted checkerboard artifacts (Table S1)^42^. We additionally adopted both short skip connections (via element-wise summation) and long skip connections (via channel-wise concatenation) to the generator to help converge the training quickly and recover the full spatial resolution (Table S1)^43^. Especially in the short connection, we used a max-pooling layer to emphasize the local maximum in learning a residual representation of the input data. For the 3D model, we concatenated the volumetric sparse localization OR-PAM image and the volumetric regular OR-PAM image and used them as input to the generator to compensate for the vascular structure lacking in the sparse localization-based images^30,33^. On the other hand, for PACT, the performance was rather poor due to the difference in the spatial resolutions of the regular and sparse images, so the corresponding dense localization-based image was fed as the target into the generator. Our discriminators consist of five convolutional layers connected in series and contain approximately 5 million trainable parameters for the 3D network and 1.5 million trainable parameters for the 2D network (Fig. S3 and Table S2). The dense localization-based image and the image synthesized from the generator were used as inputs for the discriminator. It is worth mentioning that we first trained our 2D network with localization OR-PAM maximum amplitude projection (MAP) images, and then we fine-tuned the network using the localization PACT dataset to compensate for the relatively small amount of data in PACT compared to OR-PAM. We incorporated the training strategy since the two angiographic datasets share similar feature spaces that could provide useful guidance to the networks during training. By adopting this transfer learning technique, we could further enhance the 2D networks’ reconstruction ability^44^. While training the network, to save a checkpoint, we evaluated the network at every epoch using a validation set, which consisted of 36 segmented volumetric images with 64 × 64 × 64 pixels (for a 3D network) or 30 planar images with 896 × 1024 pixels (for a 2D network). Network training ended at 200 epochs, and the trained networks were evaluated on an independent test set.

### 3D label-free localization OR-PAM based on a 3D DNN

**Fig. 2:**
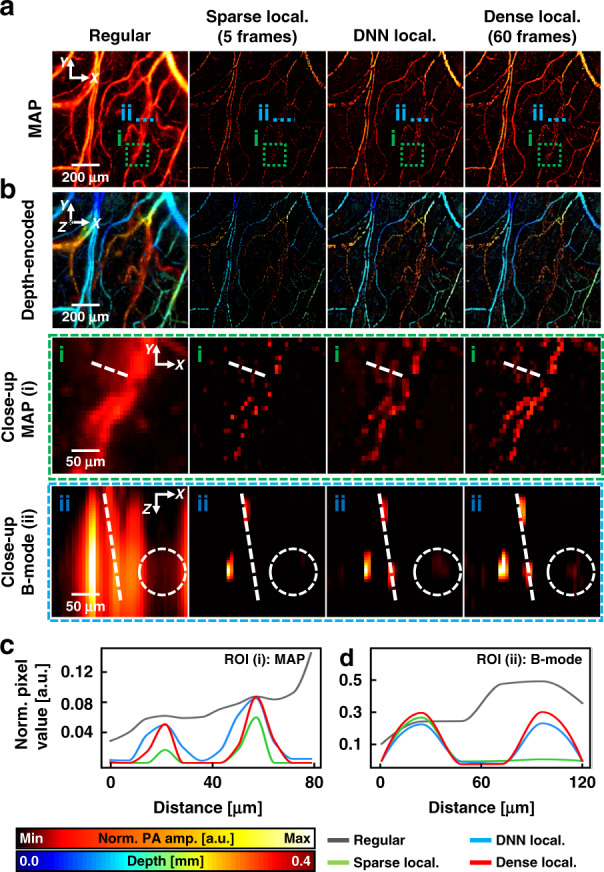
Performance of 3D deep-learning localization OR-PAM. **a** MAP and **b** depth-encoded mouse ear images of regular, sparse localization, DNN localization, and dense localization OR-PAM. Frame counts of 60 and 5 are used for the dense and sparse localization-based images, respectively. Close-up views of the regions outlined by the green dashed boxes and cross-sectional B-mode images of the region highlighted by the blue dashed lines **a** are displayed. Profiles of the PA amplitude are indicated by the white dashed lines in the close-up view of **c** MAP and **d** B-mode images, respectively. *OR-PAM* optical-resolution photoacoustic microscopy, *MAP* maximum amplitude projection, *PA* photoacoustic, *Sparse local.* sparse localization-based PA image, *DNN local.* deep neural network localization-based PA image, *Dense local. PA* dense localization-based PA image, *ROI* region of interest, *Norm. PA amp.* normalized PA amplitude

Figure 2 represents representative 3D network outputs, where regular OR-PAM images were obtained from a mouse ear in vivo, and sparse images reconstructed with the frame count of 5 were used as input. The total imaging time for the dense localization-based image was 30 s, whereas for the sparse image, it was just 2.5 s (Fig. 2a, b). The DNN localization OR-PAM images consist of 12 segmented volumetric images measuring 64 × 64 × 64 pixels along the x, y, and z axes, respectively. In Fig. 2a, we display PA MAP images with an amplitude-based color map that enables comparing PA amplitude profiles. Additionally, Fig. 2b shows PA MAP images represented with a depth-encoded color map^45^.

The 3D structural information is well-inherited from the volumetric sparse images, thanks to the 3D operations in our DNN. To emphasize the reconstruction ability of our trained network for producing 3D volumetric superresolution OR-PAM images, we enlarged the region outlined by the green dotted boxes “i“ in Fig. 2a, which include two adjacent micro blood vessels. It is qualitatively observed that the sparse localization-based MAP image has a lower signal-to-noise ratio (SNR) and sparser vessel connectivity than the dense and generated DNN images. Furthermore, the line profiles of the regions indicated by the white dashed lines in the magnified images are qualitatively comparable between the DNN MAP images (Fig. 2c). The two adjacent blood vessels are clearly resolved in the DNN and dense localization-based images, whereas they are not in the regular OR-PAM image. The profile from the sparse image indicates a lower SNR.

To demonstrate the advantage of using our 3D networks to reconstruct volumetric superresolution OR-PAM images, we also extracted B-scan images in the regions highlighted by the blue dashed lines “ii“ in Fig. 2a (Fig. 2d). The profiles were measured in the regions highlighted by the white dashed lines in the B-mode images. Similar to the profiles in the MAP images, the unbranched blood vessels in the regular PA image are well distinguished in the profiles of the DNN and dense localization-based images. Notably, a blood vessel in the sparse image is invisible, whereas the same blood vessel is revealed with high contrast in the DNN localization-based image. Also note that our network helps visualize vessel connectivity. A blood vessel highlighted by the white dashed circles, in which the sparse image has a low SNR, is well-restored in the DNN localization-based image. Even though the sparse image does not contain the vessels, they are restored in the DNN localization-based image because our network is based on 3D convolutions, allowing for the reference of adjacent pixels in 3D space. These results prove that our DL-based framework can reconstruct a dense 3D super-resolved OR-PAM image from a sparse one, and can reduce the imaging time for an agent-free localization OR-PAM image by a factor of 12 (Movie S1).

**Fig. 3:**
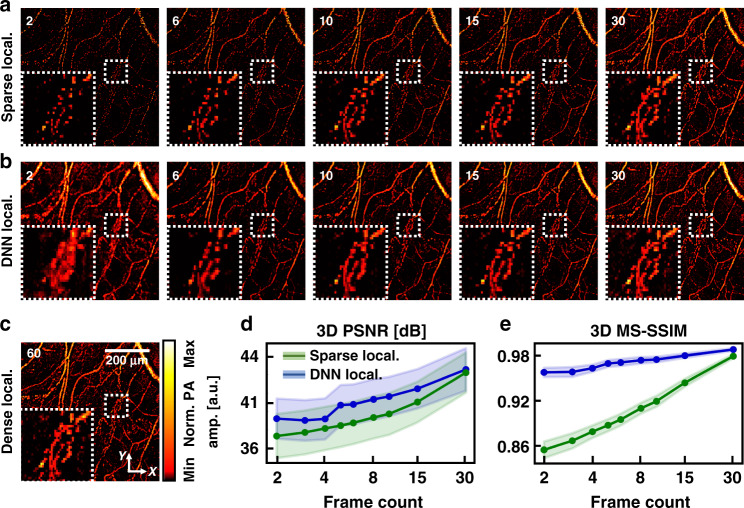
Performance of 3D deep-learning localization OR-PAM depending on frame counts. **a** Sparse localization OR-PAM images reconstructed with 2, 6, 10, 15, and 30 frames. **b** DNN localization OR-PAM images generated from sparse images. **c** A dense localization OR-PAM image reconstructed with 60 frames. All images correspond to images in Fig. 2. Graphs for **d** 3D PSNR and **e** 3D MS-SSIM evaluation metrics for frame counts of 2, 3, 4, 5, 6, 8, 10, 15, and 30. *OR-PAM* optical-resolution photoacoustic microscopy, *PA* photoacoustic, *Sparse local.* sparse localization-based PA image, *DNN local.* deep neural network localization-based PA image, *Dense local.* dense localization-based PA image, *Rep.* repetition count, *Norm. PA amp.* normalized PA amplitude, *PSNR* peak signal-to-noise ratio, *MS-SSIM* multiscale structural similarity

The number of frames used for the reconstruction of agent-free 3D localization OR-PAM images directly determines the quality of the superresolution localization-based image. We prepared training, validation, and test datasets with 2, 3, 4, 5, 6, 8, 10, 15, and 30 frames to compare the output qualities and trained nine generator networks for the 3D localization OR-PAM (Fig. 3). Each trained generator was applied to the test set, including 240 segmented volumetric images with pixel counts of 64 × 64 × 64 along the *x*, *y*, and *z* axes, respectively, which were reconstructed with a frame count corresponding to the training set. The results are summarized in Fig. 3. The sparse localization-based images are reconstructed with frame counts of 2, 6, 10, 15, and 30 (Fig. 3a), and their corresponding DNN localization-based images (Fig. 3b) are displayed. A dense localization-based image was reconstructed with a frame count of 60 (Fig. 3c). For the input frame count of 2, the overall blood vessel structures are well-restored, but the blood vessels are clumped in the enlarged image. As the frame count increases, the clumped vessels disappear, and the DNN localization-based images become similar to the dense localization OR-PAM image. Additionally, the 3D peak signal-to-noise ratio (PSNR) and 3D multiscale structural similarity (MS-SSIM) between the DNN or sparse images and the dense images were calculated with frame counts of 2, 3, 4, 5, 6, 8, 10, 15, and 30 (Fig. 3d, e)^46^. Both the PSNR and MS-SSIM increase with the number of repetitions (Fig. 3d). A PSNR value of 40.70 dB and MS-SSIM of 0.97 are achieved at a frame count of 5 for the DNN localization-based images, while corresponding metrics for the sparse images are 38.47 and 0.89, respectively. Our network achieved MS-SSIM values of above 0.98 for input frame counts above 10.

To demonstrate the extrapolation ability of our trained networks on datasets with various numbers of frames, we compared the evaluation metrics (3D PSNR and 3D MS-SSIM) obtained with all combinations of the frame counts of trained networks and sparse images (Fig. S4a, b and Table S3). In each column containing the scores obtained with various counts of frames of the sparse images and fixed counts of frames of the trained networks, the top three scores are bolded in green. Scores lower than that of the sparse images are bolded in red. Note that the test dataset with 30 frames does not always enhance the image quality in each column, because the input images are already perceptually similar to the ground truth. Both metrics have high values in cases where the frame count of the dataset used in training is similar to that of the test dataset image, which follows intuitively. Although scores with a large difference between the frame counts of the training and test sets were lower than the score of the input image, network results were further improved in most combinations. The results demonstrate that our DNN framework can improve the quality of a sparse image, even if the quality of the sparse image used for training differs from that of an actual input image to be tested. Thus, to some extent, our 3D DNNs can extrapolate to data not included in the training dataset.

### 2D labeled localization PACT based on a 2D DNN

**Fig. 4:**
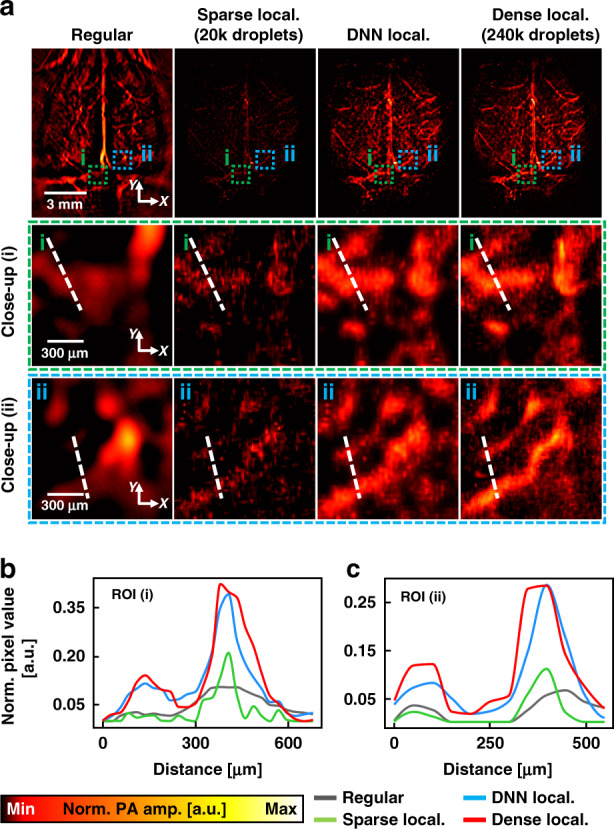
Performance of 2D deep-learning localization PACT. **a** Regular, sparse localization, DNN localization, and dense localization PACT images of a mouse brain. Droplet counts of 240,000 and 20,000 are used for the dense and sparse localization-based images, respectively. Close-up views of the regions outlined by the **i** green and **ii** blue dashed boxes in **a** are displayed. Profiles of the PA amplitude indicated by the dashed lines in (**b**) **i** and (**c**) **ii** images, respectively. *PACT* photoacoustic computed tomography, *PA* photoacoustic, *Sparse local.* sparse localization-based PA image, *DNN local.* deep neural network localization-based PA image, *Dense local.*, dense localization-based PA image, *ROI* region of interest, *Norm. PA amp.* normalized PA amplitude

Representative 2D network results, including regular PACT, sparse localization-based, DNN localization-based, and dense localization-based images, are displayed in Fig. 4, where regular PACT images were obtained from a mouse brain in vivo. The dense localization-based image was reconstructed with 240,000 dye droplets, whereas 20,000 droplets were used to generate the sparse localization-based image measuring 896 × 1024 pixels along the *x* and *y* axes, respectively (Fig. 4a). Obtaining the dense localization PACT image took half an hour^25^, but only 2.5 min were required to acquire the sparse PACT image. We enlarged the two areas indicated by the green and blue dotted boxes in Fig. 4a to observe the synthetic ability of our network in detail. The connectivity of blood vessels can be compared in the magnified images: it is difficult to recognize the vascular morphology in the regular and sparse localization-based images, whereas the DNN and dense images exhibit microvasculatures. Furthermore, we obtained the profiles of the regions indicated by the white dotted lines in the magnified images to qualitatively compare the improvement (Fig. 4b, c). The graphs for the DNN and dense localization PACT images depict two blood vessels not captured in the regular and sparse images. The amplitudes of the blood vessels in the DNN and dense localization-based images are also larger than those in the regular and sparse images, which means that the network can provide a higher SNR and contrast than the sparse image. These results suggest that our DL-based framework can provide the super-resolved PACT image 12× faster than a conventional method (Movie S2).

**Fig. 5:**
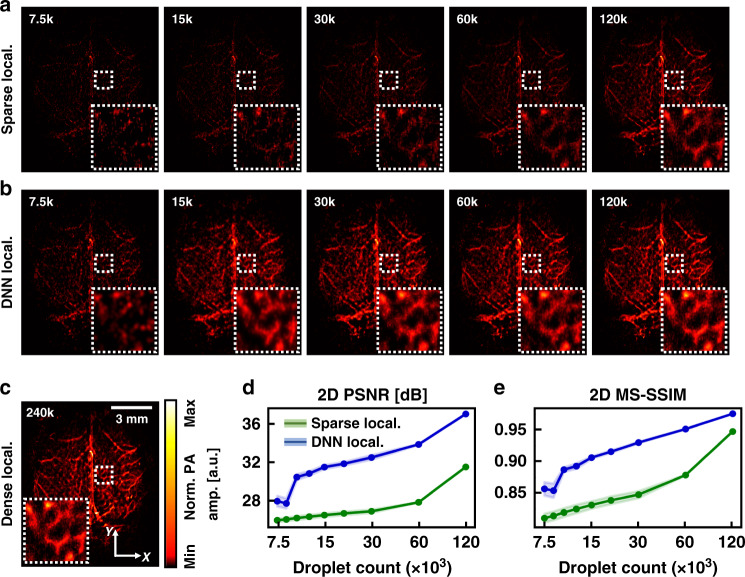
Performance of 2D deep-learning localization PACT as a function of droplet counts. **a** Sparse localization PACT images reconstructed with droplet counts of 7.5k, 15k, 30k, 60k, and 120k. **b** DNN localization PACT images generated from parse images. **c** A dense localization PACT image reconstructed with 240k droplets. All images correspond to images in Fig. 4. Graphs for **d** 2D PSNR and **e** 2D MS-SSIM evaluation metrics for droplet counts of 7.5k, 8.6k, 10k, 12k, 15k, 20k, 30k, 60k, and 120k. *PACT* photoacoustic computed tomography, *PA* photoacoustic, *Sparse local.* sparse localization-based PA image, *DNN local.* deep neural network localization-based PA image, *Dense local.*, dense localization-based PA image, *Norm. PA amp.* normalized PA amplitude, *PSNR* peak signal-to-noise ratio, *MS-SSIM* multiscale structural similarity

To investigate the effect of the number of droplets on the quality of output images synthesized by our DL network, as in the study on localization OR-PAM, we used various numbers of droplets (i.e., 1/32, 1/28, 1/20, 1/24, 1/16, 1/12, 1/8, 1/4, and 1/2 of the dense images’ droplet counts) (Fig. 5). Each trained generator was applied to the test set, consisted of 200 planar images measuring 896 × 1024 pixels along the *x* and *y* axes, respectively, reconstructed with a droplet count corresponding to that of the training set. Sparse localization-based images used as input were reconstructed with droplet counts of 7.5k, 15k, 30k, 60k, and 120k (Fig. 5a), and the corresponding DNN localization-based images as output are synthesized (Fig. 5b). A dense localization PACT image reconstructed with a droplet count of 240k is displayed (Fig. 5c). For a more detailed comparison, we zoomed in on a specific area in each image. Although the droplet count of 7.5k shows poor qualitative comparisons to the dense localization-based image, it is confirmed that the sparse images with droplet counts of above 15k were restored similarly to the dense image. Additionally, we compared the 2D PSNR and 2D MS-SSIM evaluation metrics to quantify the ability of the 2D networks (Fig. 5d, e). As the droplet count of the sparse image increases, the localization PACT image becomes denser, and thus the PSNR and MS-SSIM increase. The results demonstrate that our DL-based framework can reconstruct high-quality superresolution localization PACT images within a much shorter imaging time than typical localization PACT imaging.

Similar to the extrapolation study in localization OR-PAM, we compared the PSNR and MS-SSIM evaluation metrics with various droplet counts of the sparse images used for network training and the test set (Figs. S4c, d and Table S4). The top three values in each column are bolded in green, and output scores lower than the input are bolded in red. Contrary to the results from localization OR-PAM, the test datasets with high numbers of droplets show high scores in most columns. Most of the generated outputs also produced higher evaluation metric values than sparse images, proving the extrapolation ability of the 2D network. A possible reason for the improved generalizability performance compared to the 3D network is that we incorporated a transfer learning strategy when training the 2D networks^44^. Thus, the 2D networks were trained with datasets from a broader range of feature spaces (localization OR-PAM and PACT), enabling improved generalizability performance. The results demonstrate that our 2D networks are robust to data variations regarding the localized droplet count.

## Discussion

For use with label-free OR-PAM and labeled PACT, we introduce fast localization-based PA imaging based on a DL method that reduces the need for large numbers of images. Conventional localization methods for both OR-PAM and PACT achieve super-resolved microvasculature images by continuously imaging a target and then localizing the absorber (i.e., RBCs for label-free OR-PAM and dye droplets for labeled PACT). However, consecutive imaging slows down temporal resolution, limiting the widespread use of the technique in preclinical and clinical applications requiring fast imaging. The realized DL-based framework synthesizes dense localization OR-PAM/PACT images from sparse reconstructed ones with tens of times fewer frames or dye droplets than used in conventional dense images. Our framework can reduce the data acquisition time by 12-fold for both localization OR-PAM (MS-SSIM > 0.97) and localization PACT (MS-SSIM > 0.92). These results demonstrate that our technique could dramatically enhance the temporal resolution of both superresolution localization OR-PAM and PACT without qualitative sacrifices.

In detail, the framework consists of two subnetworks, which are developed with 2D and 3D layers, respectively, to cover both label-free volumetric localization OR-PAM images and labeled planar localization PACT images. Each subnetwork is adapted from the pix2pix framework, whose generator is based on the U-net architecture^38,40^. In the training process of the 2D network, the localization OR-PAM MAP images were first used as input because of the relatively small amount of data in PACT compared to OR-PAM. After pre-training with the localization OR-PAM dataset, the network was fine-tuned with the localization PACT dataset, a process called transfer learning^44^. This training method allowed us to train the 2D networks successfully with relatively small amounts of PACT data.

Prior to our work, DNNs have been utilized in superresolution localization fluorescence microscopy (i.e., PALM and STORM) to accelerate the localization imaging process by reducing the total number of frames and localizations that are required to reconstruct a superresolution localization image^30^. However, our work differs in that PAI is scalable from microscopy to CT, covering images on scales from micro to millimeters. Thus, our framework can extend to preclinical/clinical applications on various scales. Furthermore, we have shown feasibility for not only 2D image data but also for 3D volume structure (OR-PAM) by designing 3D convolutional neural networks, which has not been demonstrated in previous works.

An important caveat in our framework is the limited memory size of the graphical processing unit (GPU). Preprocessing is necessary because our DNNs use preprocessed sparse localization OR-PAM or PACT images as input, rather than using regular images. For the 3D network, we use 3D convolutional layers to keep 3D structural information intact; therefore, 3D volumetric images are used as input. However, 3D images contain many more pixels than 2D images, and in addition, 3D convolutional kernels store more trainable parameters than 2D kernels. Therefore, a sparse localization-based image is used as an input to synthesize a dense localization-based image, instead of using multiple regular OR-PAM images. For localization PACT, a total of 36,000 regular PACT images are used to synthesize dense localization-based images, and at least 1125 images are used to synthesize sparse localization-based images. Because using regular PACT images as input will overflow the GPU memory, we instead use a preprocessed sparse localization-based image as input. Localization preprocessing is also cumbersome and time-consuming, and the framework can be much more user-friendly if regular PA images are used as input instead of sparse localization-based images. Using an auxiliary recurrent neural network (RNN) to predict the flow positions of absorbers with a minimum number of frames might construct a framework with regular PA images as input and accelerate our framework further, a topic for future work.

Another scope of future research includes further investigating the black-box mechanism of the proposed DNNs, thus strengthening the reliability and interpretability aspect of our method. Saliency mapping algorithms (e.g., gradient-based class activation mapping^47^ and layer-wise relevance propagation^48^) can be utilized to better understand how the highly nonlinear 2D and 3D convolutional filters operate to reconstruct dense images from sparse. Such studies could shed valuable insight to design a DNN model that is more robust to problems such as false blood flow generation.

Although our initial study was conducted with OR-PAM images of mouse ears and PACT images of mouse brains, we believe that our established networks could, to a certain extent, extrapolate to similar angiographic data since microvascular profiles share morphological analogies between similar sample types and structures (e.g., mammal retina, ear, brain, and subcutaneous microvessels)^10,14^. Therefore, we aim to continually refine our DL frameworks’ generalizability by training with more images from various in vivo sample types and angiographic structures. Furthermore, by combining our established framework with transfer learning techniques^44^, acquiring a large amount of data required for retraining can be circumvented.

By reducing the image count needed in localization-based PA methods, our DL framework enhances the promising potential of existing in vivo label-free localization OR-PAM and labeled localization PACT. This framework provides superresolution PA images tens of times faster than conventional methods, so it can be used to study phenomena such as immediate drug responses that cannot be observed with conventional localization methods. For superresolution OR-PAM images, dense localization-based images are synthesized with the intact 3D structural information of sparse localization OR-PAM images. One practical result is that this new method can be used in diagnosing skin conditions and skin diseases, such as skin tumors, warts, and fungal infections that require accurate structural information. Utilizing the framework can also significantly reduce the irradiated laser and imaging time, reducing the subject’s burden during imaging. In addition, it also increases the potential utility of localization PA imaging in neuroscience, monitoring brain hemodynamics and neuronal activity. The improved temporal resolution makes high-quality monitoring possible by sampling at a higher rate, allowing analysis of fast changes that cannot be observed with conventional low temporal resolution.

## Materials and methods

### Volumetric localization OR-PAM image acquisition and preprocessing

Volumetric image data were obtained from a galvanometer scanner OR-PAM system (OptichoM, Opticho, South Korea), shown in Fig. S5. The system imaged a region of interest (ROI) in a mouse ear over two hundred times. The obtained volumetric data measured 256 pixels along the *z* axis, with a pixel size of 3 μm. The pixel sizes along the *x* and *y* axes were 3.75 μm and 5 μm, respectively. To use GPU memory efficiently, we reduced the number of pixels in the axial direction by four times with bicubic downsampling and antialiasing in the B-mode images. Considering the theoretical axial resolution limit of over 114 μm for OR-PAM systems, this reduction increased the training efficiency of the 3D DL networks, which had limited GPU memory (Supplementary Materials and Methods). Our previously reported agent-free localization imaging process was used in the current work (Supplementary Text)^10^. As in the previously reported study, volumetric localization OR-PAM images were reconstructed from 60 frames randomly selected from the obtained data. The reconstructed image, called a dense localization OR-PAM image, is the target for training and ground truth for evaluation. A corresponding regular OR-PAM image was randomly selected among the 60 images. Using the same imaging process, a corresponding sparse localization OR-PAM image was reconstructed with *k* < 60 randomly selected images among 60 images. Regular, sparse localization, and dense localization OR-PAM images were paired. To standardize the image pixel size, we cropped the volume images with different pixel dimensions to 150 × 150 × 64 pixels. Before being fed into our DNNs, the volumetric localization OR-PAM images were augmented by random cropping to a size of 64 × 64 × 64 pixels and random flipping in the *x* and *y* axes (with a flip probability of 0.5). A total of ~3000 pairs were prepared.

### Planar localization PACT image acquisition and preprocessing

RF signals acquired from the 512-channel DAQ systems were first jitter-corrected by using the PA signals from the surfaces of the ultrasonic transducer elements as reference timings (Fig. S7). The conventional PACT images were constructed using the dual-speed-of-sound universal back-projection algorithm, with a pixel size of 25 μm^49^. To trace injected dye droplets in the brain, we applied our previously reported algorithm to the conventional PACT images, precisely localizing the center of each droplet (Supplementary Text)^25^. Adding up all the *N* droplets yielded a superresolution image, called a dense localization PACT image, defined as the target for training and the ground truth. Among the *N* droplets, *k* droplets (*k* < *N*) were randomly selected to reconstruct a sparse localization PACT image. A pixel size of 5 μm was used in the superresolution image reconstruction. The sparse and dense localization PACT images were paired. To mimic localization OR-PAM MAP images and accommodate the transfer learning process, the PACT images were reduced from 2000 × 2400 pixels to 896 × 1024 pixels. The images were cropped to 512 × 768 pixels for the training set to utilize only regions with rich vascular profiles and flipped in the *x* and *y* axes (with a flip probability of 0.5) for augmentation. A total of ~500 pairs were prepared.

### Artificial neural network

The suggested framework is customized from the pix2pix architecture^40^, a special conditional GAN for image-to-image problems. The framework consists of two distinct DNN models: (1) a 3D model built with 3D operations for volumetric OR-PAM images, and (2) a 2D model built with 2D operations for planar PACT images. Although each model employs different dimensions of operations, their architectures are unified (Figs. 1, and S3 and Tables S1, S2). Each model includes a generator network *G* and a discriminator network *D*. The generator network *G*, adapted from U-net, consists of an encoder network (downsampling blocks in Fig. 1) and a decoder network (up-sampling blocks in Fig. 1). Each network is further presented in Fig. 1c and Table S1. In the 3D model, the encoder takes two-channel images, including a regular OR-PAM image and a sparse localization OR-PAM image. In contrast, a sparse localization PACT image is fed into the encoder in the 2D model. Each model adopts different up-sampling methods: transposed convolution for 3D, and pixel shuffle for 2D^42^. In the 2D model, the spatial dropout^50^ and batch normalization^51^ layers were omitted because the operations deteriorated the results (Table S1). The discriminator network consists of four convolution blocks in series using the leaky rectified linear unit^52^ function as the main activation function and an output convolution layer with a sigmoid activation function (Fig. S3 and Table S2).

DL training is generally performed by minimizing the objective function (also called the loss function). We designed our loss functions using an adversarial training scheme consisting of a generator network *G* and a discriminator network *D*, which we optimized in an alternating manner to solve the adversarial min-max problem and boost the reconstruction performance:

1 $$\mathop {{\min }}\limits_G \mathop {{\max }}\limits_D E_{y\sim P_{data\left( y \right)}}\left[ {logD\left( y \right)} \right] + E_{x\sim P_{data(x)}}\left[ {\log \left( {1 - D\left( {G\left( x \right)} \right)} \right)} \right]$$

where *x* denotes the sparse localization PAI image used as input, and *y* denotes the corresponding dense PAI image used as the ground truth. The idea is that we train our generator network *G* to fool the discriminator that distinguishes the reconstructed PAIs from their dense localization counterparts. The adversarial training strategy allows our generator network *G* to create perceptually superior images residing in the manifold of the real dense PAIs. The adversarial loss function for our 3D localization OR-PAM network is defined as follows:

2 $$L^{3D} = 0.01 \times \frac{1}{N}{\sum} {\left| {y - G\left( x \right)} \right| + \left( { - logD\left( {G\left( x \right)} \right.} \right)}$$

where *N* denotes the number of pixels in each OR-PAM image. We implemented the loss function by combining the mean absolute error (MAE) with the adversarial loss instead of the mean squared error, which yields poor results in image-to-image translation tasks^39,53^. For the 2D localization PACT network, we additionally incorporated the MS-SSIM loss because it better preserved the contrast in high-frequency regions^53^. The pre-training loss function for the transfer learning process is defined as follows:

3 $$L^{2D\_TL} = 0.3 \times \frac{1}{N}{\sum} {\left| {y - G\left( x \right)} \right| + 0.7 \times \left( {1 - MSSSIM\left( {y,G\left( x \right)} \right)} \right)}$$

where *TL* denotes transfer learning, and the *MSSSIM* function calculates the corresponding metric. After pre-training the generator networks, we further trained the networks with the PACT dataset, using the full adversarial loss defined as follows:

4 $$\begin{array}{l}L^{2D} = 0.03 \times \frac{1}{N}{\sum} {\left| {y - G\left( x \right)} \right| + 0.07 \times \left( {1 - MSSSIM\left( {y,G\left( x \right)} \right)} \right)} \\\qquad+ - logD\left( {G\left( x \right)} \right)\end{array}$$

The MS-SSIM loss was not used when training 3D networks: using only the MAE loss provided better results with stable performance. All trainable parameters were initialized using the He normal initialization method^52^ and optimized using the Adam optimizer^54^. In addition, an L2 regularization technique was incorporated to avoid overfitting the network parameters^55^. To set model checkpoints, we calculated the MS-SSIM metrics of the validation set during training. All hyper-parameters, including the loss function coefficients, were searched using a grid search approach and were found sufficient for all established networks (Table S5). All networks were implemented using Python 3.8.3 with a PyTorch backend. The 3D localization OR-PAM network training was conducted on NVIDIA RTX 3090 GPUs and an Intel®Core™ i9-10900X CPU. The 2D localization PACT network training was conducted on an NVIDIA TITAN Xp GPU and an Intel®Core™ i5-8400 CPU.

### PAI of animals in vivo

For OR-PAM, animal procedures in all experiments followed the regulations of the National Institutes of Health Guide for the Care and Use of Experimental Animals, with permission from the Institutional Animal Care and Use Committee of Pohang University of Science and Technology (POSTECH). During PAI, female Balb/c mice, 3–8 weeks old, were anesthetized by inhalation of 4% isoflurane gas at a 1.0 L/min flow rate. A silicone heating pad under the mouse kept the animal’s body warm. The imaging experiments used a 532 nm wavelength laser with a pulse energy of 10 mJ/cm^2^_,_ less than the ANSI safety limit of 20 mJ/cm^2^. Before imaging, hair was removed with a depilatory agent to maximize the PA signal. The ultrasonic gel was applied between the polyvinyl chloride membrane of the water tank and the ear of the mouse to match the impedances between the ear and the ultrasonic transducer. For PACT, all experimental procedures were conducted according to laboratory animal protocol (IA20-1737) approved by the Institutional Animal Care and Use Committee of the California Institute of Technology. In PACT animal experiments, 6–8 weeks old female mice (Swiss Webster, Invigo) were used. The left carotid artery of the mouse was cannulated with a polytetrafluoroethylene catheter, through which the droplet suspension was injected to administer droplets into the brain. The cannulation procedure followed the protocol reported previously^56^. Before brain imaging, the hair on the mouse head was removed by depilatory cream, and the scalp was cut open, but the skull was kept intact. During in vivo imaging, the mouse was fixed on a lab-made animal holder with its cortical plane oriented horizontally and was anesthetized by 1.5% isoflurane at an airflow rate of 1 L/min. The temperature of the mouse was regulated ~38 degrees. A piece of plastic Saran™ wrap was used to seal the bottom of the full-ring ultrasonic transducer array, and the chamber was filled with water for acoustic coupling. The mouse was placed under the water chamber of the imaging system, and US gel was applied between the skull and the plastic wrap for acoustic coupling. The holder was then lifted until the brain’s cortical layer was in the focal plane of the transducer array. The maximum light fluence on the surface of the animal was ~30 mJ cm^−2^, which is below the American National Standards Institute safety limit at 780 nm.

## Supplementary information

Supplemental materials

Supplementary Movie S1

Supplementary Movie S2

## Data Availability

All data are available within the Article and Supplementary Files or available from the authors upon request.

## References

[1] Wang LV, Hu S (2012). Photoacoustic tomography: in vivo imaging from organelles to organs. Science.

[2] Jeon S (2019). Review on practical photoacoustic microscopy. Photoacoustics.

[3] Jeon S (2017). In vivo photoacoustic imaging of anterior ocular vasculature: a random sample consensus approach. Sci. Rep..

[4] Kim H (2020). PAExM: label-free hyper-resolution photoacoustic expansion microscopy. Opt. Lett..

[5] Baik JW (2020). Super wide-field photoacoustic microscopy of animals and humans in vivo. IEEE Trans. Med. Imaging.

[6] Kim JY (2015). Fast optical-resolution photoacoustic microscopy using a 2-axis water-proofing MEMS scanner. Sci. Rep..

[7] Wong TTW (2017). Label-free automated three-dimensional imaging of whole organs by microtomy-assisted photoacoustic microscopy. Nat. Commun..

[8] Shi JH (2019). High-resolution, high-contrast mid-infrared imaging of fresh biological samples with ultraviolet-localized photoacoustic microscopy. Nat. Photonics.

[9] Yao JJ (2015). High-speed label-free functional photoacoustic microscopy of mouse brain in action. Nat. Methods.

[10] Kim J (2019). Superresolution localization photoacoustic microscopy using intrinsic red blood cells as contrast absorbers. Light. Sci. Appl..

[11] Baik JW (2021). Intraoperative label-free photoacoustic histopathology of clinical specimens. Laser Photonics Rev..

[12] Ahn J (2021). High-resolution functional photoacoustic monitoring of vascular dynamics in human fingers. Photoacoustics.

[13] Cho SW (2021). High-speed photoacoustic microscopy: a review dedicated on light sources. Photoacoustics.

[14] Park J (2021). Quadruple ultrasound, photoacoustic, optical coherence, and fluorescence fusion imaging with a transparent ultrasound transducer. Proc. Natl Acad. Sci. USA.

[15] Lin L (2018). Single-breath-hold photoacoustic computed tomography of the breast. Nat. Commun..

[16] Park B (2021). 3D wide-field multispectral photoacoustic imaging of human melanomas in vivo: a pilot study. J. Eur. Acad. Dermatol. Venereol..

[17] Na, S. et al. Massively parallel functional photoacoustic computed tomography of the human brain. *Nat. Biomed. Eng.* 1–9 (2021). 10.1038/s41551-021-00735-8 PMC8630100 34059809

[18] Kim J (2021). Multiparametric photoacoustic analysis of human thyroid cancers in vivo. Cancer Res..

[19] Choi W (2018). Clinical photoacoustic imaging platforms. Biomed. Eng. Lett..

[20] Yao JJ, Wang LV (2013). Photoacoustic microscopy. Laser Photonics Rev..

[21] Yao JJ (2014). Photoimprint photoacoustic microscopy for three-dimensional label-free subdiffraction imaging. Phys. Rev. Lett..

[22] Betzig E (2006). Imaging intracellular fluorescent proteins at nanometer resolution. Science.

[23] Rust MJ, Bates M, Zhuang XW (2006). Sub-diffraction-limit imaging by stochastic optical reconstruction microscopy (STORM). Nat. Methods.

[24] Danielli A (2014). Label-free photoacoustic nanoscopy. J. Biomed. Opt..

[25] Zhang PF (2019). In vivo superresolution photoacoustic computed tomography by localization of single dyed droplets. Light. Sci. Appl..

[26] Dean-Ben XL, Razansky D (2018). Localization optoacoustic tomography. Light. Sci. Appl..

[27] Vilov S, Arnal B, Bossy E (2017). Overcoming the acoustic diffraction limit in photoacoustic imaging by the localization of flowing absorbers. Opt. Lett..

[28] Choi W, Kim C (2019). Toward in vivo translation of super-resolution localization photoacoustic computed tomography using liquid-state dyed droplets. Light. Sci. Appl..

[29] Zhao HX (2021). Deep learning enables superior photoacoustic imaging at ultralow laser dosages. Adv. Sci..

[30] Ouyang W (2018). Deep learning massively accelerates super-resolution localization microscopy. Nat. Biotechnol..

[31] DiSpirito A (2021). Reconstructing undersampled photoacoustic microscopy images using deep learning. IEEE Trans. Med. Imaging.

[32] Wang HD (2019). Deep learning enables cross-modality super-resolution in fluorescence microscopy. Nat. Methods.

[33] Nehme E (2020). DeepSTORM3D: dense 3D localization microscopy and PSF design by deep learning. Nat. Methods.

[34] Qiao C (2021). Evaluation and development of deep neural networks for image super-resolution in optical microscopy. Nat. Methods.

[35] Milecki L (2021). A deep learning framework for spatiotemporal ultrasound localization microscopy. IEEE Trans. Med. Imaging.

[36] Masutani EM, Bahrami N, Hsiao A (2020). Deep learning single-frame and multiframe super-resolution for cardiac MRI. Radiology.

[37] Brady SL (2021). Improving image quality and reducing radiation dose for pediatric CT by using deep learning reconstruction. Radiology.

[38] Ronneberger, O., Fischer, P. & Brox, T. U-Net: convolutional networks for biomedical image segmentation. In: Proceedings of the 18th International Conference on Medical Image Computing and Computer-Assisted Intervention. Munich: Springer, 234–241 (2015).

[39] Goodfellow, I. J. et al. Generative adversarial nets. In: Proceedings of the 27th International Conference on Neural Information Processing Systems. Montreal: MIT Press, 2672–2680 (2014).

[40] Isola, P. et al. Image-to-image translation with conditional adversarial networks. In: Proceedings of 2017 IEEE Conference on Computer Vision and Pattern Recognition. Honolulu: IEEE, 5967–5976 (2017).

[41] Vu T (2021). Deep image prior for undersampling high-speed photoacoustic microscopy. Photoacoustics.

[42] Shi, W. Z. et al. Real-time single image and video super-resolution using an efficient sub-pixel convolutional neural network. In: Proceedings of 2016 IEEE Conference on Computer Vision and Pattern Recognition. Las Vegas: IEEE, 1874–1883 (2016).

[43] Drozdzal, M. et al. The importance of skip connections in biomedical image segmentation. In: Proceedings of the 1st International Workshop on Deep Learning in Medical Image Analysis. Athens. Greece: Springer, 179–187 (2016).

[44] Raghu, M. et al. Transfusion: understanding transfer learning for medical imaging. *Adv. Neural Inf. Process. Syst.* **32**, 3347–3357 (2019).

[45] Cho S (2020). 3D PHOVIS: 3D photoacoustic visualization studio. Photoacoustics.

[46] Wang, Z., Simoncelli, E. P. & Bovik, A. C. Multiscale structural similarity for image quality assessment. In: Proceedings of the Thrity-Seventh Asilomar Conference on Signals, Systems & Computers. Pacific Grove: IEEE, 1398–1402 (2003).

[47] Selvaraju, R. R. et al. Grad-CAM: visual explanations from deep networks via gradient-based localization. In: Proceedings of 2017 IEEE International Conference on Computer Vision. Venice: IEEE, 618–626 (2017).

[48] Bach S (2015). On pixel-wise explanations for non-linear classifier decisions by layer-wise relevance propagation. PLoS One.

[49] Li L (2017). Single-impulse panoramic photoacoustic computed tomography of small-animal whole-body dynamics at high spatiotemporal resolution. Nat. Biomed. Eng..

[50] Srivastava N (2014). Dropout: a simple way to prevent neural networks from overfitting. J. Mach. Learn. Res..

[51] Ioffe, S. & Szegedy, C. Batch normalization: accelerating deep network training by reducing internal covariate shift. In: Proceedings of the 32nd International Conference on International Conference on Machine Learning. Lille, France: PMLR, 448–456 (2015).

[52] He, K. M. et al. Delving deep into rectifiers: surpassing human-level performance on imagenet classification. In: Proceedings of 2015 IEEE International Conference on Computer Vision. Santiago, Chile: IEEE, 1026–1034 (2015).

[53] Zhao H (2017). Loss functions for image restoration with neural networks. IEEE Trans. Comput. Imaging.

[54] Kingma, D. P. & Ba, L. J. Adam: a method for stochastic optimization. In: Proceedings of the 3rd International Conference on Learning Representations. San Diego, 2015.

[55] Goodfellow I, Bengio Y, Courville A (2016). Deep Learning..

[56] Feng J (2015). Catheterization of the carotid artery and jugular vein to perform hemodynamic measures, infusions and blood sampling in a conscious rat model. J. Vis. Exp..

