# Supplementary material for: Deep learning acceleration of multiscale superresolution localization photoacoustic imaging
Source: Light Sci Appl. 2022 May 12;11:131. doi: 10.1038/s41377-022-00820-w (PMC9095876; doi:10.1038/s41377-022-00820-w)
Supplement: Supplementary file 1 — Supplemental materials [file 41377_2022_820_MOESM1_ESM.pdf]

Supplementary Information for

Deep Learning Acceleration of Multiscale Superresolution Localization Photoacoustic Imaging

Jongbeom Kim, Gyuwon Kim, Lei Li, Pengfei Zhang, Jin Young Kim, Yeonggeun Kim, Hyung Ham Kim, Lihong V. Wang*, Seungchul Lee* and Chulhong Kim*
*Corresponding author. Email: chulhong@postech.edu, seunglee@postech.ac.kr and LVW@caltech.edu

This file includes:

Supplementary Text
Supplementary Materials and Methods
Fig. S1. Label-free localization-based OR-PAM imaging process.
Fig. S2. Labeled localization-based PACT imaging process.
Fig. S3. Customized 2D and 3D discriminator network architecture.
Fig. S4. 3D Graphs of evaluation metrics depending on frame/droplet counts used to reconstruct a sparse localization-based image for the training and test set.
Fig. S5. Configuration of an optical-resolution photoacoustic microscopy system.
Fig. S6. Spatial resolution of an optical-resolution photoacoustic microscopy system.
Fig. S7. Configuration of a photoacoustic computed tomography system.
Table S1. Summary of generator networks.
Table S2. Details of the discriminator networks.
Table S3. Comparison of 3D PSNR and 3D MS-SSIM metrics depending on frame counts used to reconstruct a sparse localization-based image for the training and test set.
Table S4. Comparison of 2D PSNR and 2D MS-SSIM metrics depending on droplet counts used to reconstruct a sparse localization-based image for the training and test sets.
Table S5. Hyper parameters for training.

Movie files:

Movie S1. Formation of sparse, DNN, and dense localization-based OR-PAM images.
Movie S2. Formation of sparse, DNN, and dense localization-based PACT images.

Supplementary Text:

Label-free localization optical-resolution photoacoustic microscopy (OR-PAM)

We adopted a previously-reported localization processing procedure to reconstruct label-free super-resolution OR-PAM images (Fig. S1)[1]. The localization processing was applied to OR-PAM volumetric images obtained by a galvanometer scanner OR-PAM (OptichoM, Opticho, South Korea). The system is described in Fig. S5. The OR-PAM system, with a fast temporal resolution (B-scan speed of 500 Hz), could capture intrinsic red blood cells (RBCs) instantaneously, generating photoacoustic (PA) signals from their locations (Fig. S1). Because each frame captured the signals in the flowing blood at different points, a densely-connected localization image could be reconstructed from multiple OR-PAM frames obtained while continuously imaging the same region of a mouse ear. Finally, localization frames were translated from OR-PAM frames by localizing the PA signals within the frames, and then were superimposed to create a dense localization-based OR-PAM image.

Labeled localization photoacoustic computed tomography (PACT)

A previously-reported localization algorithm for PACT was applied to produce datasets for a 2D deep neural network (Fig. S2)[2]. A PACT system (Fig. S7) continuously imaged the cortical layer of a mouse brain during injection of small dyed droplets that had a higher optical absorption contrast than RBCs at the optical wavelength of 780 nm. Thanks to the higher contrast, the droplets could be tracked and localized with high precision. Imaging for half an hour at a frame rate of 20 Hz resulted in a total of 36,000 frames. As in the localization OR-PAM environment, the flow within the blood vessel caused each droplet to be detected at different subsequent positions. In the localization PACT processing, droplets were extracted from the acquired PACT images, and then all the localized droplets were combined to produce a dense localization-based PACT image (Fig. S2).

Supplementary Materials and Methods:

Label-free localization OR-PAM

The regular OR-PAM images for training were obtained using an OR-PAM system (Fig. S5) with an optical fiber to deliver optical pulses onto the target. Thanks to the fiber, we could obtain volumetric images from a fixed target, reducing motion artifacts during the imaging experiments. To excite a target, the system uses a fast nano-pulse laser system with a maximum pulse repetition rate of 600 kHz (VPFL-G-10, Spectra-Physics, USA). The laser beams are collimated with a fiber optic collimator (TC25FC-543, Thorlabs, USA) and then focused by an objective lens (LA1213-A, Thorlabs, USA) (Figs. S5a, b). The laser beams pass through the center hole of a customized ring-shaped ultrasound transducer with a focal length of 21 mm, an outer diameter of 15 mm, an inner diameter of 2.5 mm, a central frequency of 20 MHz, and a bandwidth of 60%. In this PAM system, we used a galvanometer scanner (GVS001, Thorlabs, USA), and we newly designed the mirror of the galvanometer scanner to steer optical beams downward (Fig. S5b). The focused beam is reflected by the mirror, steered by the scanner, and irradiates the target, thereby inducing PA waves. The PA waves are then reflected by the mirror of the scanner and measured by the transducer. A multifunctional data acquisition board (DAQ, NI PCIe-6321, National Instruments, USA) synchronizes all the mechanical systems (i.e., the laser system, galvanometer scanner, linear motorized stages, and the digitizer). B-scan images are obtained by fast angular scanning of the galvanometer scanner, and volumetric images are acquired by scanning of the linear motorized stage slowly during the fast angular scanning. The maximum B-mode imaging speed reached 500 Hz, with lateral and axial resolutions of 9.1 μm and of 114 μm (Fig. S6), respectively, under a scanning range of ~1.5 mm, 400 pixels, and a laser repetition rate of 400 kHz. The measured resolutions matched well with the theoretical lateral and axial resolutions of 8.5 μm and 113 μm, respectively, for an optical numerical aperture of 0.032, a central frequency of 20 MHz, and a -6dB bandwidth of 60%[3]. The measured PA signals, pre-amplified by an amplifier (ZX60-3018G-S+, Mini-Circuits, 26-dB gain, USA), are finally transferred into digital signals by the digitizer (ATS-9350, Alarzatech, USA) and saved in binary format.

Localization-based OR-PAM images were reconstructed through the following processes: Fast-acquired volumetric OR-PAM images were precisely aligned via an intensity-based image registration algorithm[4]. The aligned volume data was spatially interpolated to a size of 4x along x- and y-axes by bicubic interpolation. After being normalized and convolved with an averaging filter with a kernel size of 3 × 3 × 3 to emphasize local maximum points, the OR-PAM images were transferred into volumetric localization frames by determining the local maximum points in MATLAB. A super resolution volumetric localization OR-PAM image was then reconstructed by superimposing all the localization frames. The localization OR-PAM imaging improved the spatial resolution by a factor of 2.5 *in vivo*[1].

Labeled localization PACT

The PACT system used in this study is shown in Fig. S7. A Ti: Sapphire laser (LS-2145-LT-150, Symphotic Tii; 20 Hz pulse repetition rate; 12 ns pulse width) is used to output 780 nm pulses for PA excitation. The laser beam is first homogenized by an optical diffuser (EDC-5, RPC Photonics) and then illuminates the mouse brain from above. The PA signals are detected by a full-ring ultrasonic transducer array (Imasonic) with a 10-cm diameter, a 5-MHz central frequency, more than 90% one-way bandwidth, and 512 elements. Each element (20-mm height, 0.61-mm pitch, and 0.1-mm inter-element space) is cylindrically focused to produce an axial focal distance of 45 mm (acoustic NA, 0.2). The combined foci of all 512 elements form an approximately uniform imaging region with a 20-mm diameter and 1-mm thickness. In our experiments, a lab-made 512-channel preamplifier (26 dB gain) was directly connected to the ultrasonic transducer array housing, with minimized connection cable length to minimize cable noise. The pre-amplified photoacoustic signals were digitized using a 512-channel data acquisition system (four

SonixDAQs, Ultrasonix Medical ULC; 128 channels each; 40 MHz sampling rate; 12 bits dynamic range) with programmable amplification up to 51 dB. The digitized radio frequency data were first stored in the onboard buffer, then transferred to a computer. The digitized raw data were fed into a half-time dual-speed-of-sound universal back-projection algorithm for image reconstruction[5]. The in-plane resolution of this system was previously quantified as ~150 μm for an imaging size of 10 mm × 12 mm and a pixel size of 25 μm[6]. In the PACT localization experiments, IR-780, an iodide hydrophobic dye (425311, Sigma-Aldrich), was used as an optical contrast agent in the droplets. A mixture of 67% (v/v) clove oil (C8392, Sigma-Aldrich) and 33% (v/v) peanut oil (P2144, Sigma-Aldrich) was the solvent. The oil mixture was prepared so that the final solution had a density close to that of water, which guaranteed good stability of the droplets in both water and whole blood. It took 24–48 hours to fully dissolve the dye in the oil solvent, obtaining a maximum concentration of 2 mM. A mixture of 20 μL of the dye solution (2 mM) and 2 μL of surfactant (span® 80, S6760-250ML, Sigma-Aldrich) was added to 1 mL of distilled water and then vibrated for 10 s to form a droplet suspension. The final droplet suspension had a concentration of ~ 4×10^7 mL^{-1}.

To reconstruct a localization PACT image, a cortical layer of a mouse brain was imaged for 30 minutes with a frame rate of 20 Hz during droplet injection (Fig. S7). To trace the droplets in the brain, the time-lapse PACT images were first denoised by applying a 2-D adaptive noise-removal filter[7], then subtraction of adjacent frames highlighted moving droplets. To localize the single droplets, the differential images were converted into binary images by thresholding the pixel values at 1/4 of their maxima. The bright spots within a range of 16 to 64 pixels in the binary images were the regions containing droplets. Any spots with a roundness of less than 0.7 were abandoned, which removed droplet clusters and artifacts. The centroids of the bright spots in the binary images were determined to coarsely locate single droplets in the differential images. Then, a ROI of each droplet, centered at its centroid, was isolated from the differential images. The ROI (11 × 11 pixels) was fitted with a 2-D Gaussian function, yielding a precise localization of the center of each droplet. Every droplet was characterized by a 2-D Gaussian-distributed spot with a radius equal to its localization uncertainty. Adding up all the droplets yielded a super-resolution localization PACT image, which improved the spatial resolution by a factor of 6 *in vivo*.

Supplementary Figures:

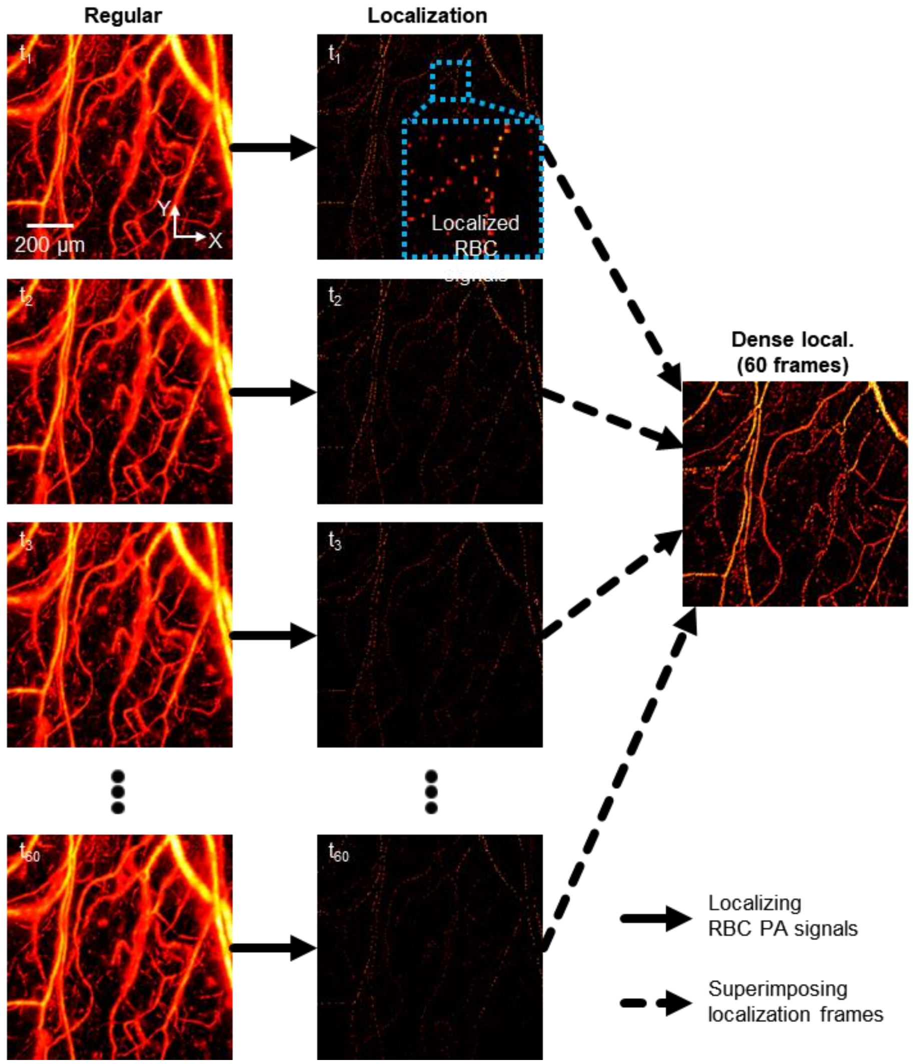

Fig. S1. Label-free localization-based OR-PAM imaging process. A regular OR-PAM frame is first translated into a localization frame, and then all the localization frames are superimposed to reconstruct a dense localization-based OR-PAM image. PA, photoacoustic; OR-PAM, optical-resolution photoacoustic microscopy; RBC, red blood cell; Dense local., dense localization-based PA image.

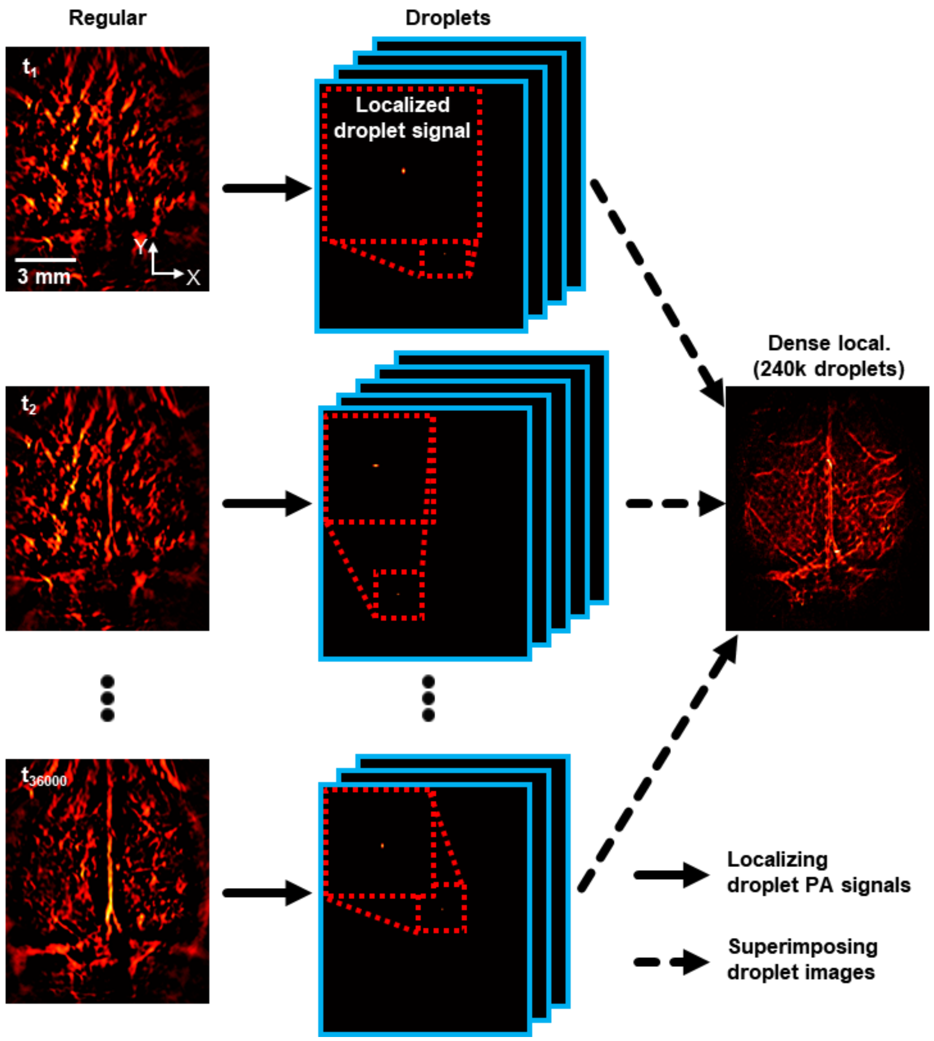

Fig. S2. Labeled localization-based PACT imaging process. Droplets containing a hydrophobic dye are intravenously injected. Each droplet is localized from regular PACT images. All the extracted droplets are combined to reconstruct a dense localization-based PACT image. PA, photoacoustic; PACT, photoacoustic computed tomography; Dense local., dense localization-based PA image.

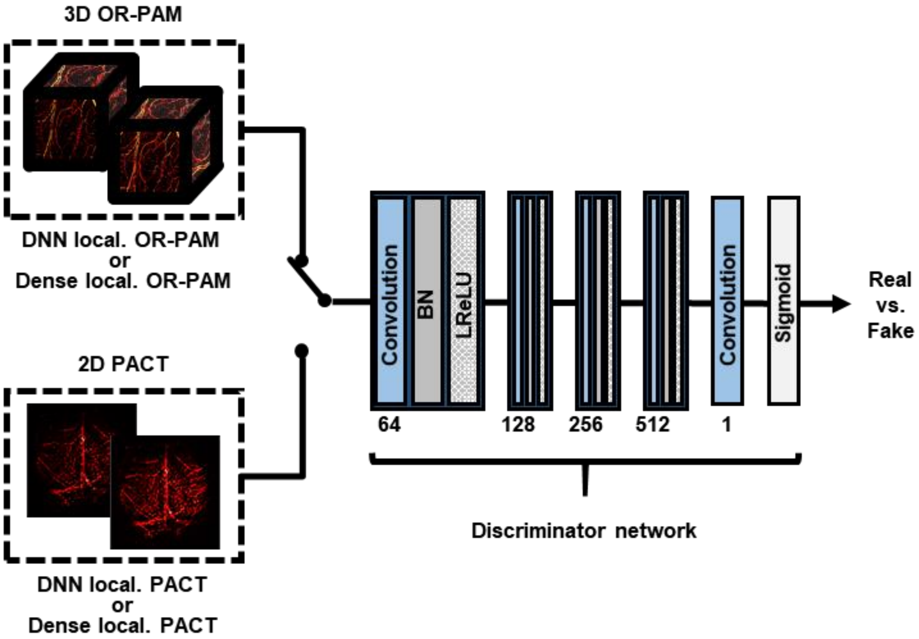

Fig. S3. Customized 2D and 3D discriminator network architecture.

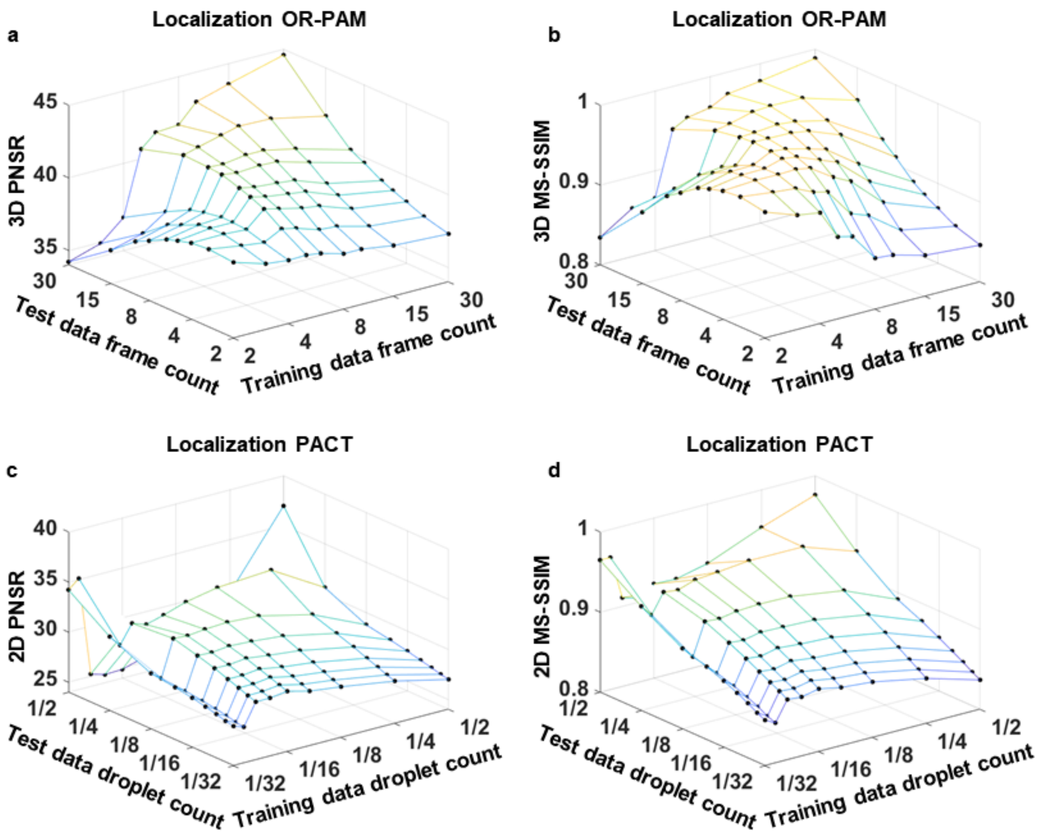

Fig. S4. 3D Graphs of evaluation metrics depending on frame/droplet counts used to reconstruct a sparse localization-based image for the training and test set. Graphs for (**a**) 3D PSNR and (**b**) 3D MS-SSIM evaluation metrics of localization OR-PAM for frame counts of 2, 3, 4, 5, 6, 8, 10, 15, and 30. Graphs for (**c**) 2D PSNR and (**d**) 2D MS-SSIM evaluation metrics of localization PACT for droplet counts of 1/32, 1/28, 1/20, 1/24, 1/16, 1/12, 1/8, 1/4, and 1/2 of the dense images' droplet counts. OR-PAM, optical-resolution photoacoustic microscopy; PSNR, peak signal-to-noise ratio; MS-SSIM, multi-scale structural similarity; PACT, photoacoustic computed tomography.

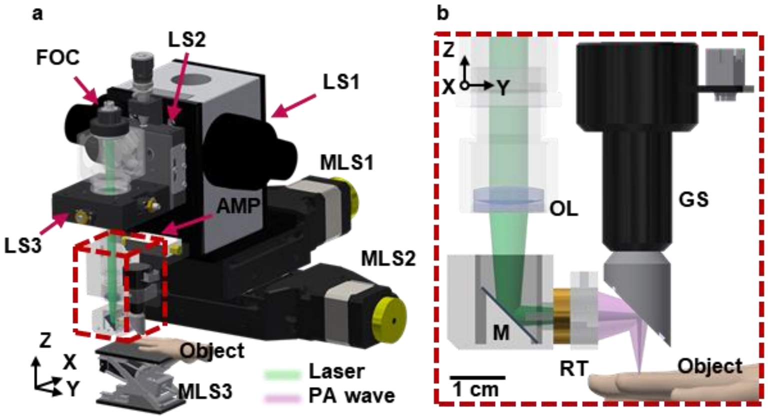

Fig. S5. Configuration of an optical-resolution photoacoustic microscopy system. (**a**) 3D model of the system. (**b**) Close-up view of the scanning part outlined by the red dashed box in (**a**). FOC, fiber optic collimator; LS, linear stage; MLS, motorized linear stage; AMP, amplifier; OL, objective lens; GS, galvanometer scanner; M, mirror; RT, ring transducer.

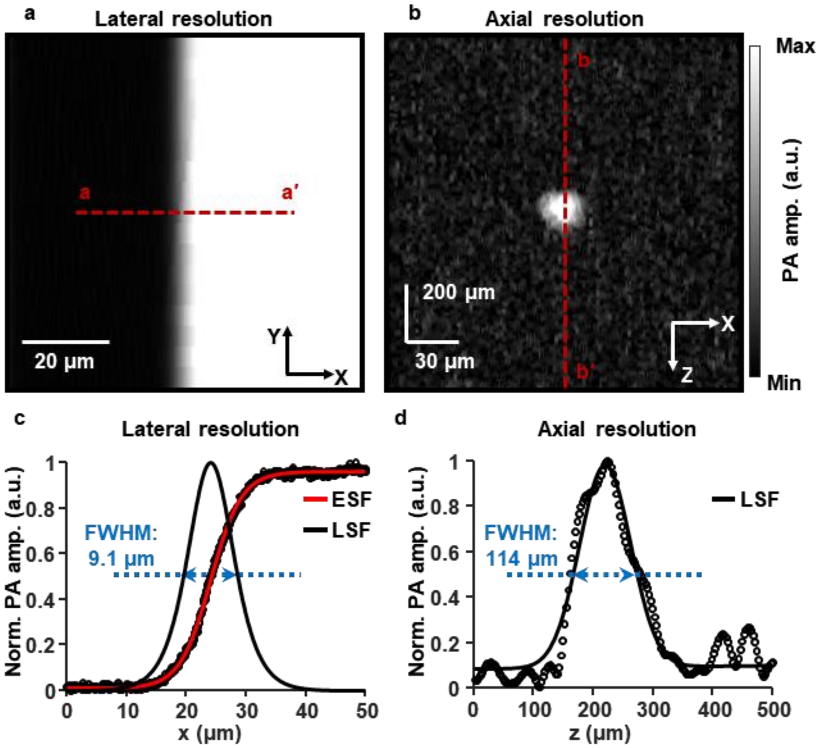

Fig. S6. Spatial resolution of an optical-resolution photoacoustic microscopy system. (**a**) PA MAP image of the edge of a patterned microstructure. (**b**) Cross-sectional PA B-scan image of a carbon fiber. (**c**) Fitted ESF of PA data marked by the line a-a′ in (**a**), and LSF, defined as the first derivative of the ESF. The lateral resolution was measured by the FWHM of the LSF. (**d**) Fitted LSF of PA data marked by the line b-b′ marked in (**b**). The axial resolution was measured by the FWHM of the LSF. PA, photoacoustic; ESF, edge spread function; LSF, line spread function; MAP, maximum amplitude projection; FWHM, full width at half maximum.

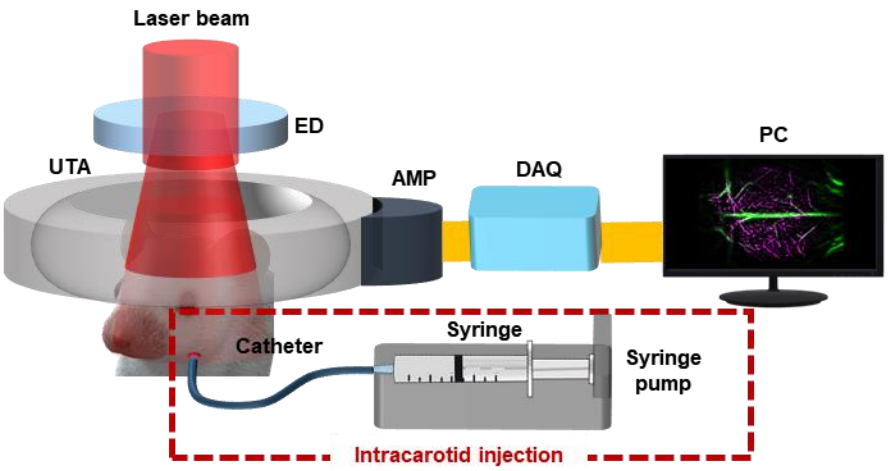

Fig. S7. Configuration of a photoacoustic computed tomography system. A mouse brain is photoacoustically imaged during intracarotid injection of a droplet suspension through a catheter. ED, engineered diffuser; UTA, ultrasonic transducer array; AMP, amplifier; DAQ, data acquisition board; PC, personal computer.

Table S1. Summary of generator networks.

U-Net generator network								
Layer		Operation	Output channels	Filter size	Stride	Padding	Output padding	Coefficient
Contraction		Convolution	64/128/256/512/512	3	2	1	-	-
		Batch normalization[a]	64/128/256/512/512	-	-	-	-	-
Short skip connection		Convolution	64/128/256/512/512	1	1	0	-	-
		Max pooling	64/128/256/512/512	2	2	0	-	-
		Batch normalization[a]	64/128/256/512/512	-	-	-	-	-
		Element-wise summation	64/128/256/512/512	-	-	-	-	-
		LReLU	64/128/256/512/512	-	-	-	-	0.2
Bridge connection		Convolution	512	3	1	1	-	-
		Batch normalization[a]	512	-	-	-	-	-
		LReLU	512	-	-	-	-	0.2
Long skip connection		Channel-wise concatenation	1024/1024/512/256/128	-	-	-	-	-
Expansion	2D	Convolution	[1024/1024/512/256/128]*4	3	1	1	-	-
		Pixel shuffle	1024/1024/512/256/128	-	-	-	-	2
		Convolution	512/256/128/64/32	3	1	1	-	-
		LReLU	512/256/128/64/32	-	-	-	-	0.2
	3D	Transposed convolution	512/256/128/64/32	3	2	1	1	-
		Batch normalization[a]	512/256/128/64/32	-	-	-	-	-
		LReLU	512/256/128/64/32	-	-	-	-	0.2
		Spatial dropout[b]	512/256/128/64/32	-	-	-	-	0.5
Output convolution		Convolution	1	3	1	1	-	-
		Sigmoid	1	-	-	-	-	0.2

[a, b] The spatial dropout and batch normalization operations were omitted in the 2D localization PACT network because they deteriorated the results.

Table S2. Details of the discriminator networks.

Discriminator network						
**Layer**	**Operation**	**Output channels**	**Filter size**	**Stride**	**Padding**	**Coefficient**
Contraction	Convolution	64/128/256/512	3	2	1	-
	Batch normalization[a]	64/128/256/512	-	-	-	-
	LReLU	64/128/256/512	-	-	-	0.2
Output convolution	Convolution	1	3	2	1	-
	Sigmoid	1	-	-	-	-

[a] The batch normalization operation was omitted in the 2D localization PACT network because the operation deteriorated the results.

Table S3. Comparison of 3D PSNR and 3D MS-SSIM metrics depending on frame counts used to reconstruct a sparse localization-based image for the training and test set. For each pre-trained network, the top 3 scores are bolded. Scores lower than the score without the network are bolded in red. (MEAN ± SD)

**3D PSNR with respect to the frame counts**										
**Test data frame count**	**Without network**	**Training data frame count**								
		2	3	4	5	6	8	10	15	30
2	37.33±2.41	**39.22±2.19**	38.54±2.30	38.37±2.36	38.41±2.25	38.27±2.29	37.83±2.21	37.83±2.21	37.50±2.30	**37.31±2.35**
3	37.72±2.39	**39.37±2.31**	39.03±2.21	38.83±2.30	39.46±2.27	39.24±2.34	38.73±2.21	38.64±2.19	38.07±2.24	37.75±2.30
4	38.16±2.36	**39.26±2.48**	**39.29±2.17**	**39.19±2.24**	40.29±2.22	40.05±2.29	39.68±2.22	39.51±2.17	38.74±2.19	38.23±2.23
5	38.47±2.33	39.00±2.60	**39.27±2.21**	**39.27±2.17**	40.70±2.19	40.46±2.24	40.24±2.21	40.08±2.15	39.25±2.15	38.59±2.16
6	38.76±2.31	**38.75±2.67**	**39.17±2.24**	**39.29±2.12**	40.98±2.16	40.78±2.18	40.65±2.20	40.52±2.14	39.72±2.15	38.93±2.14
8	39.34±2.30	**38.12±2.69**	**38.62±2.29**	**39.08±2.04**	**41.37±2.12**	**41.22±2.08**	**41.32±2.21**	**41.27±2.15**	**40.64±2.21**	39.62±2.12
10	39.73±2.28	**37.66±2.68**	**38.22±2.29**	**38.84±1.99**	**41.46±2.14**	**41.40±2.03**	**41.63±2.23**	**41.63±2.14**	**41.16±2.21**	**40.10±2.12**
15	41.03±2.23	**36.30±2.65**	**36.88±2.40**	**37.96±1.93**	**41.54±2.17**	**41.82±1.83**	**42.23±2.30**	**42.54±2.05**	**42.48±2.14**	**41.67±2.15**
30	44.24±2.19	**34.21±2.53**	**34.90±2.63**	**36.27±1.99**	**40.67±2.26**	**41.55±1.73**	**41.66±2.47**	**42.93±1.89**	**43.57±1.95**	**44.58±2.32**
**3D MS-SSIM with respect to the frame counts**										
**Test data frame count**	**Without network**	**Training data frame count**								
		2	3	4	5	6	8	10	15	30
2	0.8543±0.0110	**0.9577±0.0061**	0.9434±0.0107	0.9390±0.0113	0.9030±0.0201	0.8988±0.0203	0.8642±0.0169	0.8623±0.0146	**0.8514±0.0108**	**0.8462±0.0101**
3	0.8664±0.0102	**0.9631±0.0045**	0.9583±0.0066	0.9556±0.0078	0.9453±0.0121	0.9407±0.0131	0.9063±0.0169	0.8980±0.0147	0.8691±0.0112	**0.8561±0.0092**
4	0.8786±0.0084	**0.9608±0.0049**	**0.9634±0.0042**	**0.9632±0.0048**	0.9640±0.0057	0.9608±0.0070	0.9421±0.0084	0.9310±0.0086	0.8942±0.0089	**0.8687±0.0074**
5	0.8871±0.0081	0.9557±0.0059	**0.9625±0.0038**	**0.9631±0.0040**	0.9696±0.0046	0.9674±0.0059	0.9561±0.0075	0.9473±0.0082	0.9127±0.0100	**0.8782±0.0073**
6	0.8947±0.0079	0.9508±0.0064	**0.9605±0.0038**	**0.9614±0.0035**	**0.9727±0.0036**	0.9710±0.0047	0.9638±0.0058	0.9571±0.0066	0.9286±0.0096	**0.8881±0.0077**
8	0.9098±0.0073	0.9350±0.0083	0.9491±0.0055	0.9509±0.0047	**0.9746±0.0025**	**0.9744±0.0033**	**0.9739±0.0043**	0.9705±0.0051	0.9551±0.0075	**0.9093±0.0077**
10	0.9191±0.0073	0.9234±0.0092	0.9390±0.0067	0.9417±0.0061	**0.9731±0.0022**	**0.9742±0.0026**	**0.9769±0.0031**	**0.9749±0.0038**	**0.9656±0.0060**	**0.9239±0.0084**
15	0.9439±0.0053	**0.8894±0.0097**	**0.9081±0.0062**	**0.9136±0.0068**	0.9678±0.0021	**0.9714±0.0017**	**0.9789±0.0016**	**0.9806±0.0018**	**0.9800±0.0029**	**0.9592±0.0053**
30	0.9794±0.0027	**0.8343±0.0117**	**0.8603±0.0072**	**0.8655±0.0066**	**0.9460±0.0026**	**0.9557±0.0023**	**0.9610±0.0023**	**0.9722±0.0014**	**0.9776±0.0009**	**0.9882±0.0017**

PSNR, peak signal-to-noise ratio; MS-SSIM, multi-scale structural similarity index.

Table S4. Comparison of 2D PSNR and 2D MS-SSIM metrics depending on droplet counts used to reconstruct a sparse localization-based image for the training and test sets. For each pre-trained network, the top 3 scores are bolded in green. Scores lower than the score without the network are bolded in red. (MEAN ± SD)

**2D PSNR with respect to the droplet counts**										
**Test data frame count**	**Without network**	**Training data frame count**								
		1/32	1/28	1/24	1/20	1/16	1/12	1/8	1/4	1/2
1/32	25.97±0.19	27.95±0.56	27.60±0.53	29.79±0.43	29.81±0.38	30.03±0.50	29.46±0.42	29.07±0.38	28.23±0.37	26.99±0.31
1/28	26.06±0.19	28.11±0.56	27.72±0.53	30.07±0.37	30.09±0.32	30.29±0.43	29.77±0.37	29.38±0.35	28.49±0.35	27.15±0.31
1/24	26.20±0.20	28.39±0.57	27.96±0.54	30.43±0.31	30.43±0.27	30.64±0.36	30.16±0.32	29.78±0.31	28.86±0.35	27.40±0.32
1/20	26.34±0.21	28.65±0.57	28.16±0.53	30.82±0.25	30.81±0.24	31.01±0.29	30.61±0.26	30.25±0.27	29.27±0.34	27.66±0.33
1/16	26.51±0.19	28.94±0.49	28.38±0.45	31.31±0.18	31.28±0.18	31.48±0.20	31.19±0.20	30.85±0.23	29.80±0.34	27.98±0.31
1/12	26.69±0.19	29.23±0.49	28.59±0.44	**31.81±0.17**	**31.75±0.19**	**31.94±0.19**	**31.82±0.19**	**31.54±0.25**	**30.40±0.39**	28.34±0.34
1/8	26.90±0.18	**29.55±0.43**	**28.79±0.38**	**32.45±0.13**	**32.35±0.19**	**32.54±0.15**	**32.66±0.15**	**32.47±0.24**	**31.20±0.43**	**28.78±0.33**
1/4	27.83±0.02	**31.38±0.04**	**30.26±0.03**	**32.13±0.05**	**31.64±0.05**	**32.12±0.05**	**32.85±0.05**	**33.46±0.07**	**33.81±0.05**	**30.66±0.04**
1/2	31.49±0.03	**34.22±0.04**	**35.08±0.02**	**25.24±0.03**	**24.82±0.03**	**24.88±0.03**	**25.53±0.03**	**25.89±0.04**	**29.71±0.06**	**36.97±0.04**
**2D MS-SSIM with respect to the droplet counts**										
**Test data frame count**	**Without network**	**Training data frame count**								
		1/32	1/28	1/24	1/20	1/16	1/12	1/8	1/4	1/2
1/32	0.8106±0.0070	0.8569±0.0107	0.8501±0.0117	0.8754±0.0040	0.8739±0.0040	0.8783±0.0046	0.8728±0.0042	0.8694±0.0042	0.8562±0.0047	0.8368±0.0073
1/28	0.8142±0.0070	0.8616±0.0100	0.8541±0.0112	0.8813±0.0035	0.8798±0.0039	0.8841±0.0036	0.8793±0.0036	0.8760±0.0035	0.8628±0.0039	0.8421±0.0069
1/24	0.8196±0.0070	0.8685±0.0095	0.8606±0.0108	0.8873±0.0037	0.8856±0.0044	0.8902±0.0034	0.8859±0.0038	0.8830±0.0034	0.8703±0.0036	0.8494±0.0067
1/20	0.8250±0.0068	0.8753±0.0087	0.8665±0.0101	0.8946±0.0034	0.8928±0.0043	0.8975±0.0027	0.8940±0.0035	0.8916±0.0029	0.8792±0.0027	0.8572±0.0062
1/16	0.8314±0.0060	0.8830±0.0074	0.8731±0.0086	0.9034±0.0025	0.9016±0.0033	0.9063±0.0018	0.9039±0.0025	0.9020±0.0019	0.8902±0.0023	0.8665±0.0057
1/12	0.8388±0.0062	0.8912±0.0074	0.8798±0.0085	**0.9137±0.0020**	**0.9121±0.0027**	**0.9165±0.0015**	**0.9157±0.0020**	**0.9143±0.0016**	0.9033±0.0028	0.8776±0.0061
1/8	0.8478±0.0056	**0.9005±0.0064**	**0.8868±0.0073**	**0.9268±0.0012**	**0.9255±0.0017**	**0.9292±0.0011**	**0.9307±0.0012**	**0.9300±0.0012**	**0.9199±0.0031**	**0.8913±0.0057**
1/4	0.8786±0.0005	**0.9301±0.0005**	**0.9160±0.0006**	**0.9406±0.0004**	**0.9383±0.0005**	**0.9456±0.0004**	**0.9478±0.0004**	**0.9518±0.0004**	**0.9515±0.0004**	**0.9283±0.0006**
1/2	0.9476±0.0003	**0.9649±0.0002**	**0.9646±0.0002**	**0.9108±0.0005**	**0.9052±0.0005**	**0.9176±0.0005**	**0.9164±0.0005**	**0.9255±0.0005**	**0.9527±0.0004**	**0.9757±0.0002**

PSNR, peak signal-to-noise ratio; MS-SSIM, multi-scale structural similarity index.

Table S5. Hyper parameters for training.

Training settings				
**Type**	**Network**	**Learning rate**	**Batch size**	**Weight decay**
2D	Generator (transfer learning pre-train)	0.001	1	0.001
	Generator (GAN)	10^{-5}	1	0.001
	Discriminator (GAN)	10^{-4}	1	-
3D	Generator (GAN)	0.0025	16	0.001
	Discriminator (GAN)	0.005	16	0.001

References

1 Kim, J. *et al.* Super-resolution localization photoacoustic microscopy using intrinsic red blood cells as contrast absorbers. *Light: Science & Applications* **8**, 1-11 (2019).

2 Zhang, P., Li, L., Lin, L., Shi, J. & Wang, L. V. In vivo superresolution photoacoustic computed tomography by localization of single dyed droplets. *Light: Science & Applications* **8**, 36 (2019).

3 Yao, J. & Wang, L. V. Photoacoustic microscopy. *Laser & photonics reviews* **7**, 758-778 (2013).

4 Mathworks. *Image Processing Toolbox reference R2016a*. (MathWorks Natick, MA, 2016).

5 Xu, M. & Wang, L. V. Universal back-projection algorithm for photoacoustic computed tomography. *Physical Review E* **71**, 016706 (2005).

6 Li, L. *et al.* Single-impulse panoramic photoacoustic computed tomography of small-animal whole-body dynamics at high spatiotemporal resolution. *Nature biomedical engineering* **1**, 1-11 (2017).

7 Lim, J. S. Two-dimensional signal and image processing. *Englewood Cliffs* (1990).
